# Effect of Dataset Size and Medical Image Modality on Convolutional Neural Network Model Performance for Automated Segmentation: A CT and MR Renal Tumor Imaging Study

**DOI:** 10.1007/s10278-023-00804-1

**Published:** 2023-03-17

**Authors:** Harrison C. Gottlich, Adriana V. Gregory, Vidit Sharma, Abhinav Khanna, Amr U. Moustafa, Christine M. Lohse, Theodora A. Potretzke, Panagiotis Korfiatis, Aaron M. Potretzke, Aleksandar Denic, Andrew D. Rule, Naoki Takahashi, Bradley J. Erickson, Bradley C. Leibovich, Timothy L. Kline

**Affiliations:** 1 Mayo Clinic Alix School of Medicine, Mayo Clinic, Rochester, MN USA; 2 Division of Nephrology and Hypertension, Mayo Clinic, Rochester, MN USA; 3 Department of Urology, Mayo Clinic, Rochester, MN USA; 4 Division of Biomedical Statistics and Informatics, Mayo Clinic, Rochester, MN USA; 5 Department of Radiology, Mayo Clinic, Rochester, MN USA

**Keywords:** Kidney, Deep learning, Semantic segmentation, Nephrectomy, Machine learning model performance, Similarity metrics

## Abstract

The aim of this study is to investigate the use of an exponential-plateau model to determine the required training dataset size that yields the maximum medical image segmentation performance. CT and MR images of patients with renal tumors acquired between 1997 and 2017 were retrospectively collected from our nephrectomy registry. Modality-based datasets of 50, 100, 150, 200, 250, and 300 images were assembled to train models with an 80–20 training-validation split evaluated against 50 randomly held out test set images. A third experiment using the KiTS21 dataset was also used to explore the effects of different model architectures. Exponential-plateau models were used to establish the relationship of dataset size to model generalizability performance. For segmenting non-neoplastic kidney regions on CT and MR imaging, our model yielded test Dice score plateaus of $0.93\pm 0.02$ and $0.92\pm 0.04$ with the number of training-validation images needed to reach the plateaus of 54 and 122, respectively. For segmenting CT and MR tumor regions, we modeled a test Dice score plateau of $0.85\pm 0.20$ and $0.76\pm 0.27$, with 125 and 389 training-validation images needed to reach the plateaus. For the KiTS21 dataset, the best Dice score plateaus for nn-UNet 2D and 3D architectures were $0.67\pm 0.29$ and $0.84\pm 0.18$ with number to reach performance plateau of 177 and 440. Our research validates that differing imaging modalities, target structures, and model architectures all affect the amount of training images required to reach a performance plateau. The modeling approach we developed will help future researchers determine for their experiments when additional training-validation images will likely not further improve model performance.

## Introduction

Semantic segmentation of medical images offers new insights from standard imaging for the treatment and research of diseases. These insights may be particularly valuable for renal cell carcinoma, the eighth most common malignancy in the United States, given the frequent detection of small, potentially indolent renal masses using cross-sectional (CT) imaging [1–3]. In addition to aiding in pre-operative decision-making, readily available accurate segmentations of kidneys and tumors can be used through 3D modeling and other means to improve patient education, surgical simulation trainings, and even as overlay options in intra-operative imaging [4].

Machine learning and deep learning approaches have been used for semantic segmentation across medical specialties and specifically for renal anatomic structures [5–9].The U-Net architecture is a CNN architecture with good performance on a range of medical segmentation tasks [10–13]. With growing variations to the U-Net architecture framework, Isensee et al. published their open-sourced “no new U-Net” (nnU-Net) framework that extends the U-Net architecture by encompassing best practices for pre-processing, model selection, hyperparameter, and post-processing steps together with model architecture design [14]. The nnU-Net framework is recognized as the state-of-the-art framework in medical image semantic segmentation, being externally validated and winning several open-sourced medical image segmentation challenges in the 2018 Medical Decathlon Segmentation Challenge [15]. Additionally, all the top submissions in the 2021 open-access Kidney and Kidney Tumor Segmentation Challenges used variations of the nnU-Net framework [16, 17].

While nnU-Net is becoming established as a standard segmentation model, more work remains to study evidence-based ways to assemble datasets for robust model training. Curating representative datasets to train semantic segmentation algorithms is one of the most challenging and critical steps in model development and one that can be particularly difficult to revisit after pre-processing and modeling phases have begun. Our team sought to investigate how we can evaluate training size volume during the pre-processing and modeling steps.

Here, we hypothesize that an exponential-plateau model can be used to determine at what dataset sizes segmentation performance reaches a Dice plateau to identify when adding additional images is unlikely to improve model performance on a holdout test set with a given CNN architecture. We further investigate the generalizability of a standard nnU-Net self-adapting framework by comparing its performance on different size datasets of two different imaging modalities, CT and T2-weighted MR imaging, predicting renal tumor and non-neoplastic renal parenchyma labels. The main objective of this study is to investigate how researchers can determine the size of datasets required for automated CNN segmentation to provide robust predictions for custom medical image segmentation purposes.

## Materials and Methods

This retrospective study was approved by our institutional review board, was HIPAA compliant, and was performed in accordance with the ethical standards contained in the 1964 Declaration of Helsinki. CT and MR images from our radiology database of patients presenting with kidney tumors were collected, curated, and annotated. The final segmentations consisted of manual segmentations and automated predicted segmentations with manual correction of kidney parenchyma and renal tumors. KiTS21 images were downloaded from the open-sourced repository with details on acquisition and segmentation as described in the authors’ publication. Each image volume was used as a single case for analysis, where different slices from the same volume were not analyzed separately [18]. The details of each dataset are described in the following sections.

### CT Reference Image Segmentations

We analyzed manually segmented cross-sectional imaging derived from the previously described Nephrectomy Registry [19]. This dataset consisted of 1233 non-contrast and various contrast phase abdomen/pelvis CT images from patients who underwent a radical nephrectomy for a renal tumor between 2000 and 2017 without metastatic lesions or positive lymph nodes at the time of surgery. The scans were stored as NIfTI images with de-identified header information and corresponding manual segmentations of kidney and tumor. The manual segmentations were performed by trained medical image analysts and reviewed by an expert radiologist, nephrologist, and two urologic oncology fellows using the segmentation software ITK-snap **RRID:SCR_002010** (version 2.2; University of Pennsylvania, Philadelphia, PA) [20]. There were 356 images excluded after data curation due to having shifted voxel intensities ($n=2$), non-axial images ($n=2$), or not having manual segmentations for both kidneys and renal tumor ($n=352$). A particular characteristic of this dataset is that all the CT images were cropped around both kidneys together on the in-plane view and 3 axial slices above the most superior and 3 slices below the most inferior kidney voxels regardless of kidney laterality to reduce memory space usage in the manual segmentation process. To reduce the variability in image shape due to cropping, the scans were then resampled to a standard 256-pixel coronal plane width and 128-pixel medial plane depth. In cases where the images were smaller than the standard size, zero padding was used to reach the 256 by 128 dimension.

### MR Reference Image Segmentations

A total of 501 patients who underwent partial ($n=313$) or radical nephrectomy ($n=188$) with available MR imaging performed before surgery between 1997 and 2014 were identified from our Radiology database. Only T2-weighted with fat-saturation coronal abdominal/pelvic MR images were selected for the study ($n=419$). The images were stored in an internal server using the NIfTI format with de-identified header information. Patients with small lesions not seen on the single coronal T2-weighted MR series analyzed ($n=28$) and patients with total kidney volume (TKV) greater than 600 mL (due to polycystic kidney disease) ($n=7$) were excluded from the study. To expedite the image segmentation process, a U-Net-based algorithm trained to segment kidneys affected by polycystic kidney disease was used to segment the right and left kidneys [21]. Next, the autogenerated kidney segmentations were manually refined and the tumors were manually annotated by two urologic oncology fellows.

### Data Subsets and Stratification

To test the effect on dataset sizes, we compiled MR and CT training and validation sets of 50, 100, 150, 200, 250, and 300 reference image segmentations using an 80–20 training validation split for each fold. 50 random CT and 50 random MR reference image segmentations were separately held out for testing. For the KiTS21 data, 20% of the 300 images were held out to make a 60-image test set with the remaining 240 images available for the training-validation split. 80, 120, 160, 200, and 240 were the different training-validation set sizes that we used for the KiTS21 dataset.

### nnU-Net Specifications

For nnU-net automatic pre-processing, the correct modality was specified for each dataset as either “CT” or “T2.” nnU-Net models were trained according to the instructions on the creators’ public GitHub **RRID:SCR_002630** page [22]. Fivefold cross-validation was employed for each dataset size using the 3d_fullres configuration. For the KiTS21 experiment, both the 3d_fullres and 2D configurations were used. The final ensemble model consisted of “majority voting” of the five sub-models trained on different training and validation sets of data, where the ensemble prediction was the most common prediction from each sub-model predicting the label (background, kidney, or tumor) for a voxel.

### Segmentation Model Statistical Analysis

Final evaluation of the process was done using the different nnU-Net models to predict segmentations on the holdout modality-specific test sets. Average test Dice scores were the main metric used to compare models of different size datasets. Dice score is the most used metric in 3D imaging segmentation measuring the degree of overlap between predicted and reference standard segmentations with a perfect overlap of 1 and no overlap of 0. Taha et al. describe the Dice metric and other metrics including Jaccard, true positive rate (TPR), and mean surface distance (MSD) more fully [23]. Additionally, a paired Student’s t-test was performed between the dataset size test set predictions to evaluate the difference of model performance. The evaluation of the 300-dataset size ensemble models was done using the Jaccard index, TPR, and MSD. Further, the predicted volumes were compared to the reference standard volumes using the Bland–Altman analysis and linear regression.

### Dataset Size Performance Plateau Estimation

Segmentation model performance measured by average test Dice score was observed to plateau as dataset size increased. The exponential-plateau model was defined as

1 $$D(x) = D_M - (D_M - D_0) \times {e}^{-kx}$$

where estimated parameters are *D*_M_, the maximum achievable Dice, *D*_0_, the minimum Dice, and $k$, the exponential rate constant. $D(x)$ is the estimated Dice given $x$, the amount of training images. The parameters of the model were fit using the curve_fit function in the scipy open-sourced library that uses a non-linear least squares method [24]. We defined the plateau point as within 0.01 Dice of the maximum predicted Dice score. This model was used to investigate the relationship between dataset size and observed test Dice and to determine at what size dataset test Dice performance plateaus were reached.

## Results

### Internal Dataset

#### Patient and Image Characteristics

A total of 350 images per imaging modality were randomly selected for the study. The patient characteristics are presented in Table 1. Voxel size and slice number parameters for the CT and MR datasets can be found in Table 2. The in-plane axial resolution of CT images was standardized to 256 × 128 voxels, while MR images varied between 201 and 512 voxels with most images having a coronal in-plane resolution of 256 × 256 voxels.

**Table 1:** Cohort characteristics

Parameter	Value (CT dataset)	Value (MR dataset)
No. of subjects	350	350
Males	229	217
Females	121	133
*Age	63 ± 13 [19–88]	59 ± 14 [20–88]
*Height (m^2^)	1.72 ± 0.1 [1.43–2.04]	1.73 ± 0.1 [1.49–2.04]
*Weight (kg)	92.79 ± 25.02 [45–200]	90.17 ± 22.33 [46–190]
*BMI (kg/m^2^)	31.00 ± 7.34 [16–62]	30.13 ± 6.52 [17–57]

^*^Mean ± standard deviation

**Table 2:** Image characteristics

	CT	MR
In-plane voxel width × height (mm)		
Mean	1.03 × 1.03	1.34 × 1.34
Median	1 × 1	1.56 × 1.56
Range	0.49–1.85	0.59–1.95
Slice thickness (mm)		
Mean ± Std	4.03 ± 1.38	6.26 ± 1.65
Median	5	6
Range	0.6–8	2–15
Number of slices		
Mean ± Std	45 ± 24	32 ± 12
Median	37	30
Range	20–211	6–116

#### Kidney Segmentation Models

The best observed ensemble models for the CT and MR images were the 250 and 300 dataset size ensemble models having Dice scores of 0.93 and 0.92, respectively. The CT generally had better Dice scores at lower dataset sizes than the MR model, but the nnU-Net ensemble framework was found to provide estimates of over 0.89 mean test set Dice score for both CT and MR kidney segmentation predictions with as little as 50 training and validation examples.

**Fig. 1:**
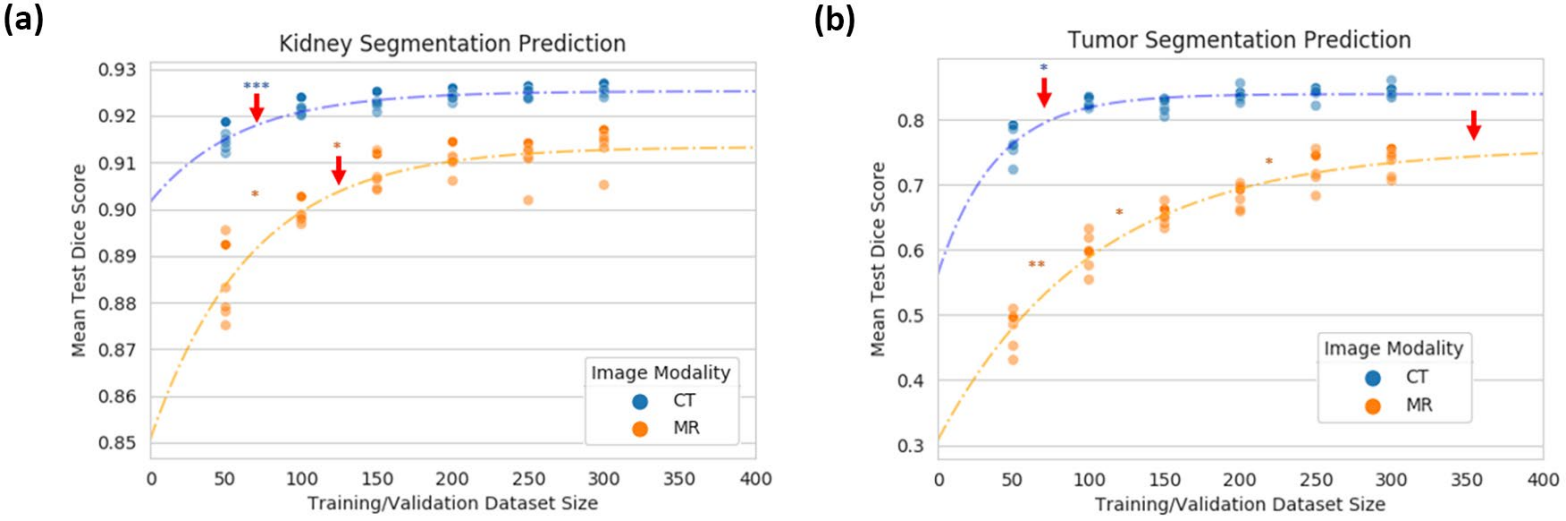
**a** Mean kidney and **b** mean tumor test Dice score prediction. The solid scatter points represent the ensemble models, and the faded color points represent the individual 5 folds at different dataset sizes. The dotted line is the fitted exponential-plateau model for CT (blue) and MR (orange) image modalities. Arrows indicate where the plateau point is estimated on each curve. Student’s paired t-test was used to determine where the ensemble predicted Dice scores for each test set were statistically significantly different, where ***$p \le 0.001$, **$p \le 0.01$, and *$p \le 0.05$

The plateau point for improved performance with more CT images from our model occurs at 54 images at an average test Dice score of 0.93, while the plateau point for MR images is at 122 images at an average test Dice score of 0.91 as seen in Fig. 1a. No statistical difference was observed between the ensemble models past the plateau point.

#### Tumor Segmentation Models

Segmenting tumors is more difficult than kidneys because of the increased heterogeneity of tumor size, shape, intensity, and the differentiation from other renal structures like simple cysts. The best performing tumor ensemble models for CT and MR were both from the 300-dataset size with average test Dice scores of 0.86 and 0.76, respectively. For segmenting tumor tissue on CT and MR, our model estimated a plateau point at 126 and 389 images at a test Dice score of 0.84 and 0.76, respectively. No statistically significant difference was observed beyond the CT tumor plateau point; however, statistical difference ($p = 0.03$; paired Student’s t-test) was observed between the 200 and 250-dataset size points for the MR tumor ensemble models. These values can be visualized in Fig. 1b. In both cases, the median test Dice scores are higher than the mean, representing the effect of difficult outlier test examples weighing down the average.

#### Stability of Plateau Prediction Analysis

**Fig. 2:**
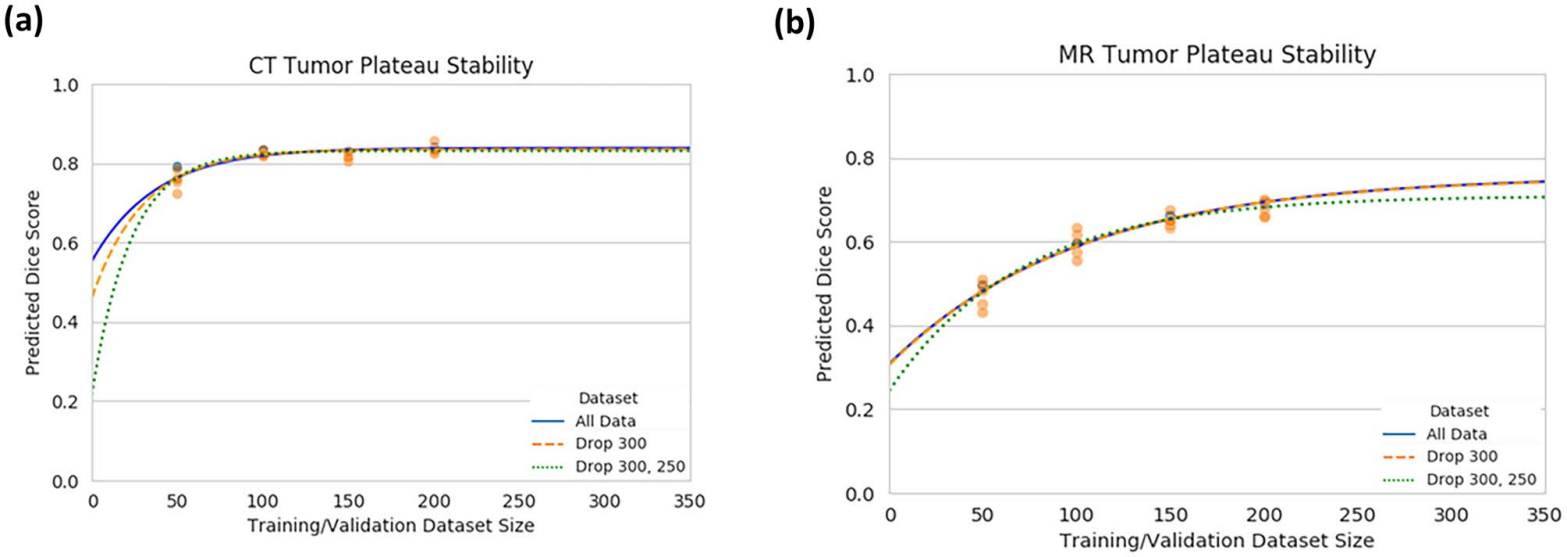
**a** Mean CT and **b** MR tumor test Dice score prediction with fitted exponential-plateau models on progressively dropped out larger datasets. The dotted lines are the fitted exponential-plateau models for all of the data (blue), dropping the 300 size dataset (orange), and dropping the 300 and 250 size datasets (green). The all data (blue) and drop 300 datasets (orange) overlap

To assess the stability of our modeling approach while simulating the usefulness of it when building a dataset, we modeled different fits dropping higher size datasets. In Fig. 2a, the reliability of the model fit is evident even without the 250 and 300 size datasets. The maximum predicted Dice for these three fits are 0.84, 0.84, and 0.83. In contrast, the plateau stability for the MR tumor is less stable with more significant differences between the three model fits. The maximal plateaus were estimated to be at a Dice of 0.76, 0.76, and 0.71 (Fig. 2b). The large jump between dropping the 300 and both dropping the 300 and 250 dataset plateaus suggests that the model is yet to stabilize, while the congruency between all the data and the drop 300 dataset plateaus suggests the model is starting to stabilize and a real performance plateau is being reached.

#### Overall Model Performance

**Fig. 3:**
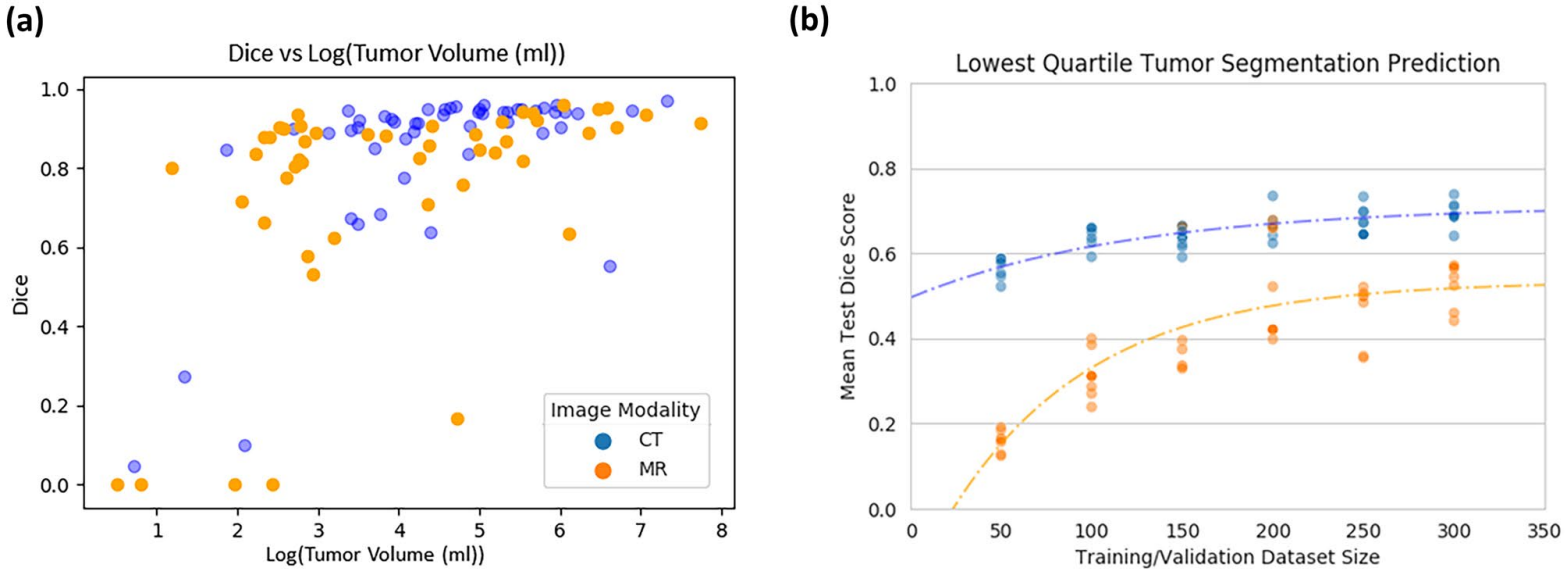
**a** Test Dice of the ensemble models trained on the 300-size dataset vs. log of tumor size for CT (blue points) and MR (orange points). **b** CT (blue) and MR (orange) exponential-plateau model fit of test Dice vs. training size of images with the lowest quartile reference segmentation tumor volumes

Looking more closely at the ensemble models from the 300-dataset size for the CT and MR models, it is evident how a few test examples have a significant effect on the overall test Dice scores with individual test scores below 0.2. These difficult examples tend to be smaller tumors, presenting more of a challenge for the model to recognize. Analysis of the CT and MR ensemble models by test Dice vs. volume is shown in Fig. 3a.

Recognizing the trend in Fig. 3, we analyzed how the test Dice of tumors in the smallest reference segmentation volume quartile in each dataset plateaued using our exponential-plateau model. The mean of CT tumor sizes in the smallest quartile of the test dataset was 22.0 ± 12.7 mL. The predicted plateau is estimated to require 378 images to reach 0.711 test Dice. For MR, the test reference segmentation volumes in the smallest quartile were all from partial nephrectomies with a mean of 8.7 ± 4.0 mL. Our model estimated a plateau at 338 images of 0.53 mean test Dice. Figure 3b shows the relationship for both the CT and MR datasets.

#### Overall Ensemble Model Test-Set Results

**Fig. 4:**
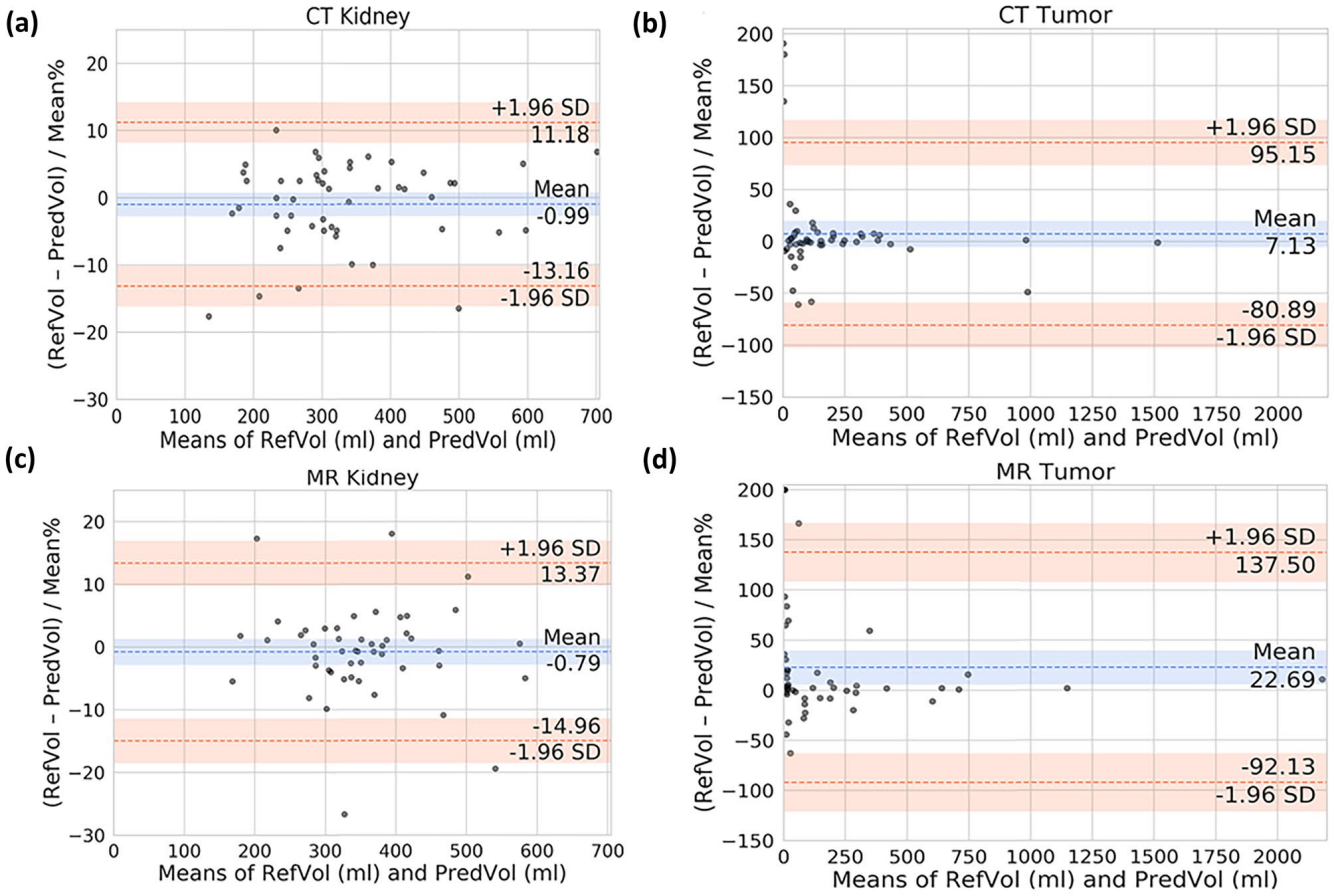
Linear regression analysis examining the concordance between predicted and reference segmentation volume sizes. **a** CT kidney. **b** CT tumor. **c** MR kidney. **d** MR tumor

We conducted a linear regression analysis for kidney and tumor volumes between the reference standard segmentations and the CT and MR ensemble model predictions. Excellent agreement is observed for the kidney segmentations with an *R*^2^ of 0.969 for the CT model and 0.904 for the MR model (Fig. 4a, c). Similarly, the tumor segmentations showed good agreement with an *R*^2^ of 0.932 and 0.982, respectively (Fig. 4b, d).

**Fig. 5:**
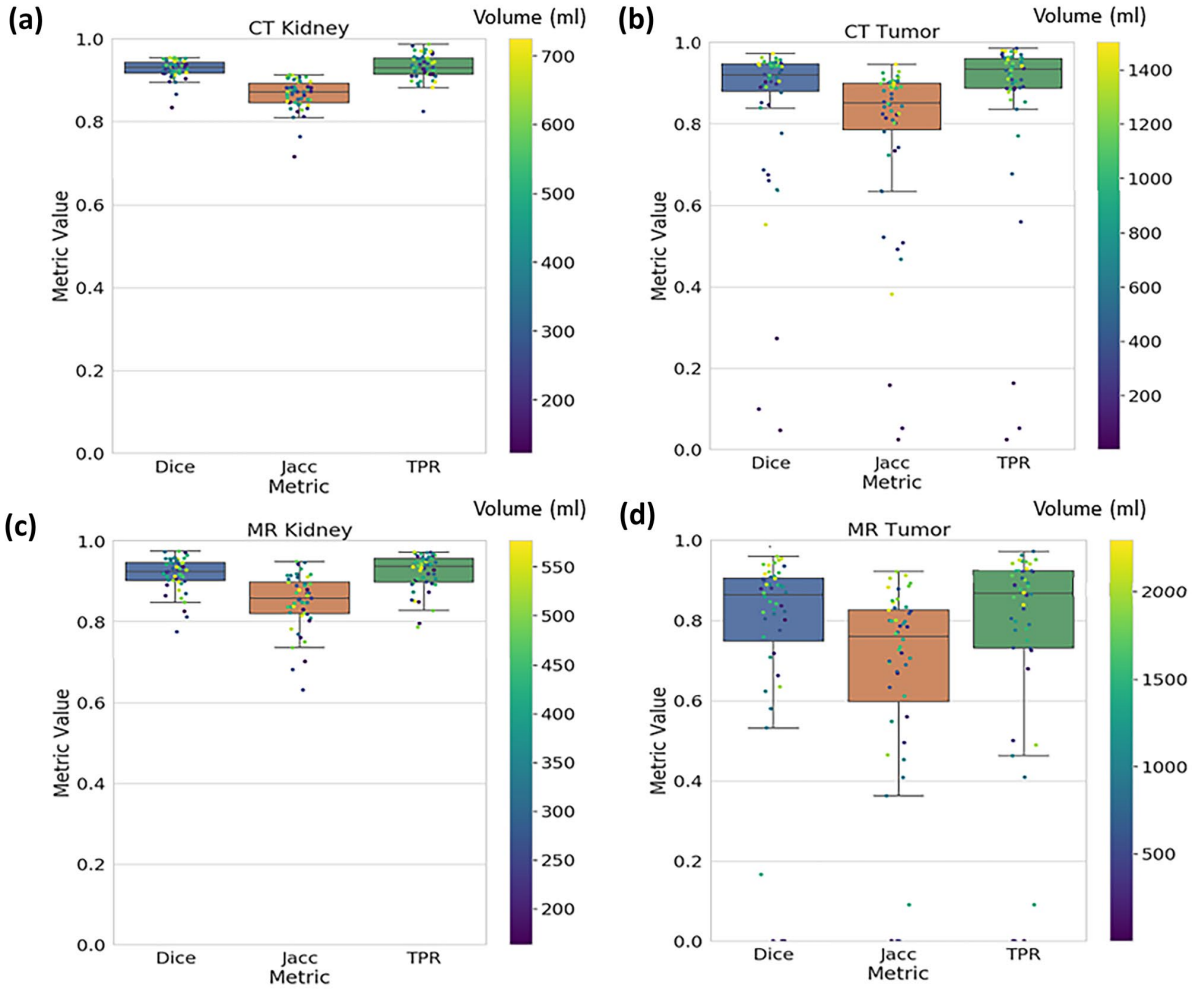
Bland–Altman analysis of final 300-dataset size model predictions for each task. **a** CT kidney. **b** CT tumor. **c** MR kidney. **d** MR tumor

Using a Bland–Altman analysis, the percent volume difference between the reference standard kidney volumes and the volumes from the CT and MR ensemble model predictions resulted in a bias ± SD of $-0.99\% \pm 6.21\%$ and $-0.79\% \pm 7.23\%$, respectively (Fig. 5a, c). A larger percent difference was observed for the case of tumor volume comparison with a bias ± SD of $6.36\% \pm 46.17\%$ and $22.69\% \pm 58.58\%$ for the CT and MR ensemble models, respectively (Fig. 5b, d).

**Fig. 6:**
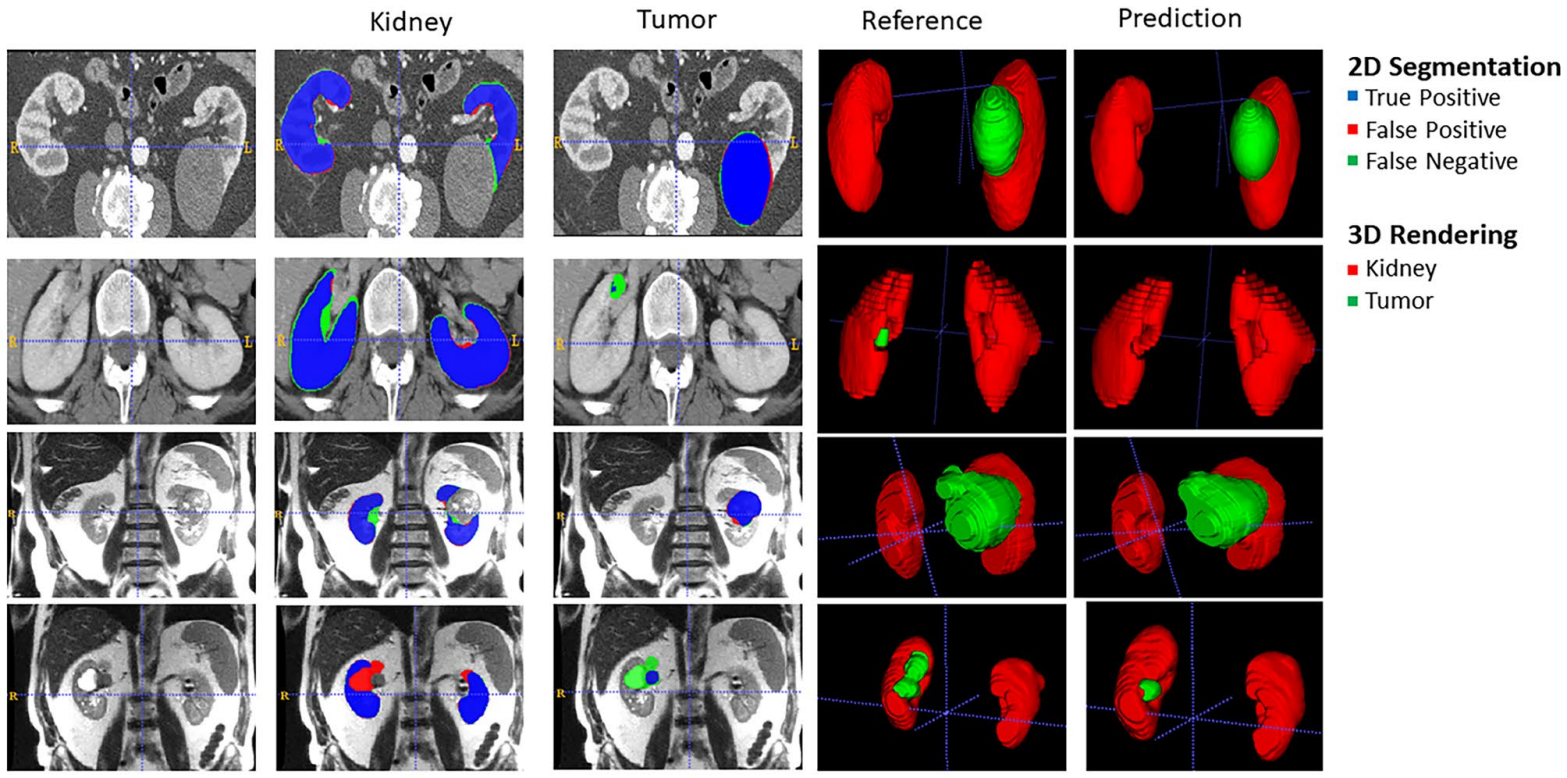
Test metrics of final ensemble 300-dataset size model predictions for each testing set example colored by reference segmentation volume. **a** CT kidney. **b** CT tumor. **c** MR kidney. **d** MR tumor

The agreement of the overlap between the reference standard and predicted segmentations was assessed by the Dice coefficient, Jaccard index (Jacc), and true positive rate (TPR). In Fig. 6, the three metrics are presented on a scale from 0 to 1 (where 1 indicates perfect agreement) evaluating the 300-dataset sizes for CT and MR ensemble model performance for kidney and tumor independently. The mean value and standard deviation of each metric distribution are summarized in Table 3.

**Table 3:** Summary statistics of the ensemble CT and MR models compared to the reference standard segmentations

Metric (mean ± SD)/image modality	Dice coefficient	Jaccard index	TPR	MSD
CT kidney	$0.93 \pm 0.02$	$0.87 \pm 0.04$	$0.93 \pm 0.03$	$0.60 \pm 0.27$
CT tumor	$0.85 \pm 0.20$	$0.77 \pm 0.22$	$0.86 \pm 0.21$	$1.42 \pm 2.12$
MR kidney	$0.92 \pm 0.04$	$0.85 \pm 0.07$	$0.92 \pm 0.05$	$0.50 \pm 0.35$
MR tumor	$0.76 \pm 0.27$	$0.66 \pm 0.26$	$0.75 \pm 0.28$	$15.15 \pm 55.54$

**Fig. 7:**
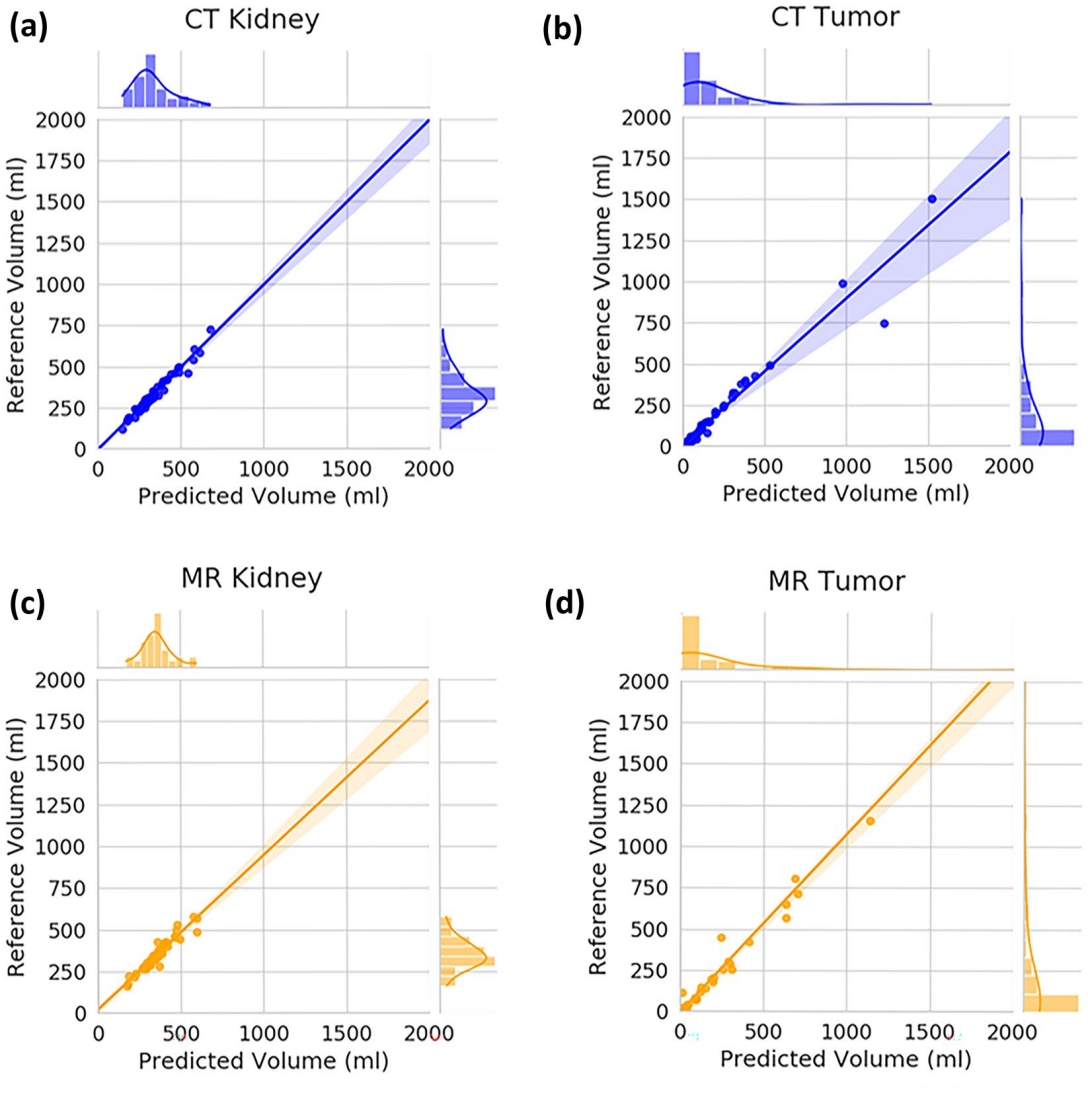
Test set image example cases illustrating both good and poor tumor model performance. **a** CT image of a large homogeneous hypointense tumor with good model prediction (kidney Dice score = 0.95, tumor Dice score = 0.96). **b** CT image of a small hypointense lesion with low contrast and not well-defined borders. The CT tumor model predicted only a small region within the tumor resulting in kidney and tumor Dice scores of 0.95 and 0.05, respectively. **c** MR image of a large heterogeneous tumor with a good model prediction (kidney Dice score = 0.96, tumor Dice score = 0.94). **d** MR image of a small hypointense tumor, in this case the MR tumor model did not generate a tumor prediction (kidney Dice score = 0.88, tumor Dice score = 0.17)

Lastly, we performed a qualitative analysis in 2D and with 3D rendering of our models to identify both good and poor performing cases as shown in Fig. 7. The false-positive label refers to voxels incorrectly predicted as kidney or tumor. The false-negative label refers to voxels incorrectly predicted as non-kidney or non-tumor. In general, we found that our model tends to produce more false-negative than false-positive segmentations, sometimes entirely missing areas of generally smaller tumors.

### KiTS21 Dataset

The KiTS21 open-sourced training dataset allowed us to further investigate the effect of different model architectures using our method with the well-established data. Notably, as described in the authors’ publication, the KiTS21 data is of all corticomedullary contrast phase images as opposed to our internal dataset that has multiple different contrast phases and non-contrast images [18]. For this dataset, we specifically investigated the performance of the different models on segmenting tumor, since we observed that even the smaller datasets were able to accurately segment non-neoplastic kidney, limiting the need to predict the dataset size saturation point for Dice performance. By focusing on just segmenting tumor, we were also able to incorporate another facet into the model investigating the effects of different model architectures, namely, comparing 2D and 3D models.

**Fig. 8:**
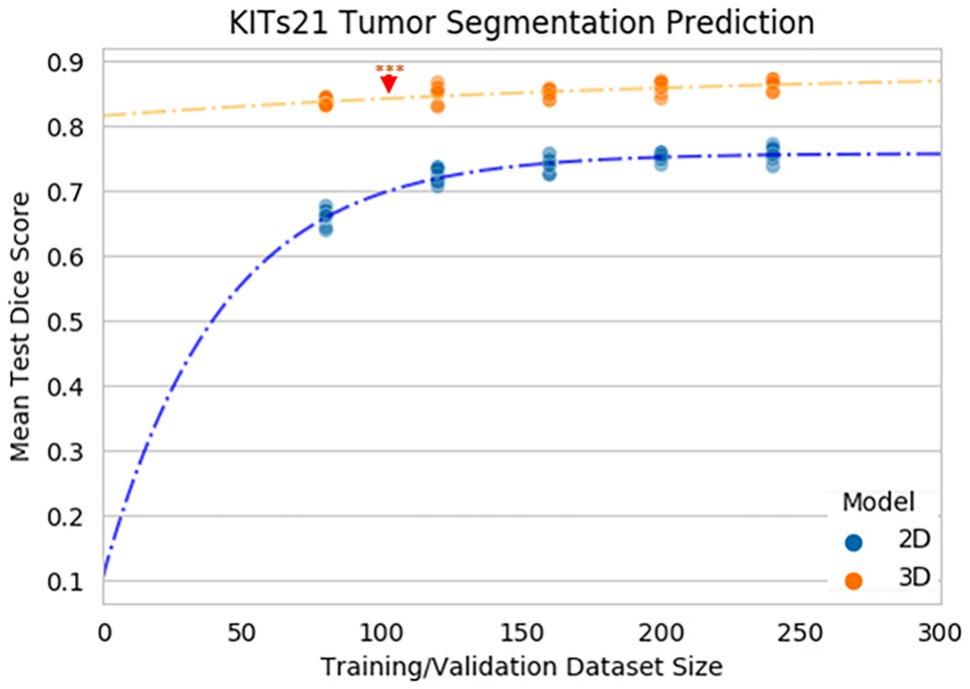
**a** Mean tumor test Dice score prediction for 2D (blue) and 3D (orange) models. The solid scatter points represent the ensemble models, and the faded color points represent the individual 5 folds at different dataset sizes. The dotted line is the fitted exponential-plateau model for 2D (blue) and 3D (orange) image modalities. Arrows indicate where the plateau point is estimated on each curve. Student’s paired t-test was used to determine where the ensemble predicted Dice scores for each test set were statistically significantly different, where ***$p \le 0.001$

Regarding performance, the top performing tumor ensemble models for the 2D and 3D architectures were both from the 300-dataset size with average test Dice scores of $0.67 \pm 0.29$ and $0.84 \pm 0.18$, respectively. The exponential-plateau model predicted a maximum with required dataset size of 0.76 at 177 images and 0.88 at 440 images for the 2D and 3D model as shown in Fig. 8a, b.

**Fig. 9:**
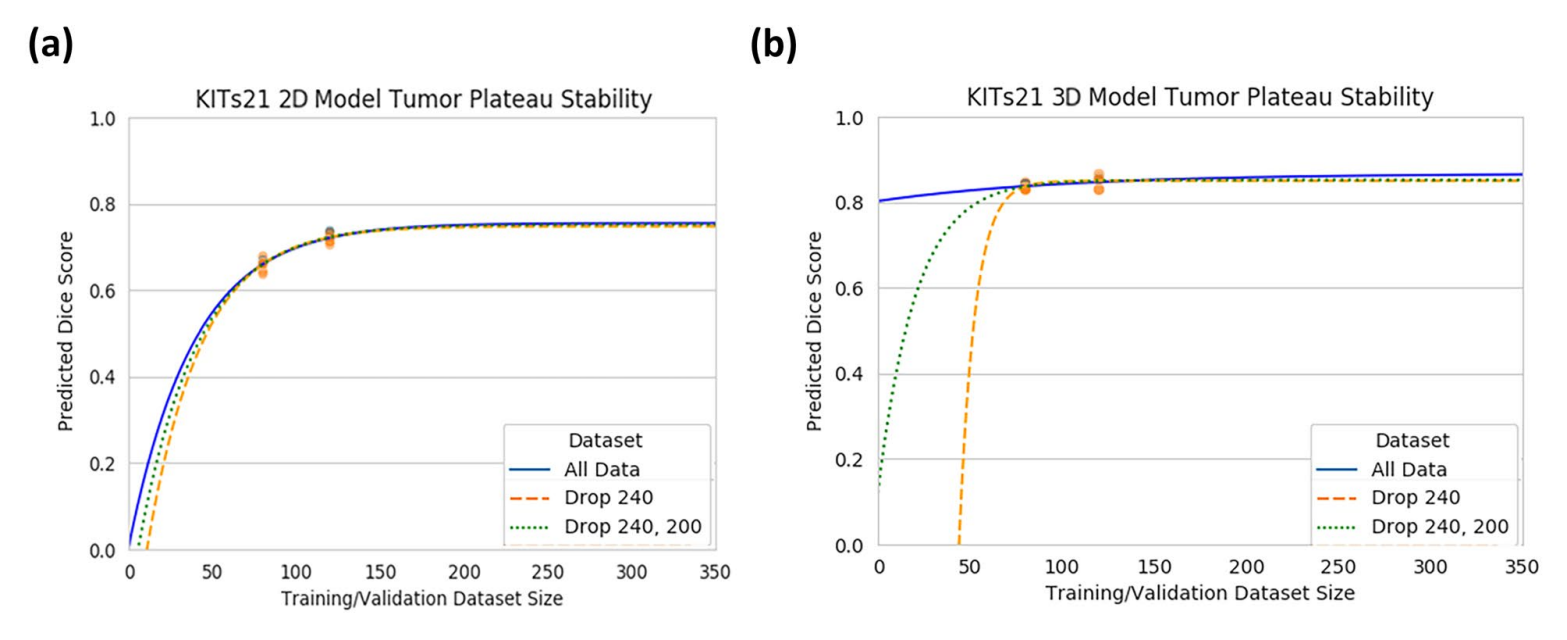
**a** Mean 2D and **b** 3D tumor test Dice score prediction with fitted exponential-plateau models on progressively dropped out larger datasets. The dotted lines are the fitted exponential-plateau models for all of the data (blue), dropping the 240 size dataset (orange), and dropping the 240 and 200 size datasets (green)

Like our experiments on the internal dataset, we investigated the stability of our plateau predictions dropping the largest size dataset sizes in the analysis. In Fig. 9a, b, the reliability of the maximum achievable Dice is evident even without the 240 and 200 size datasets. The maximum predicted Dice for the “all data,” “drop 240,” and “drop 240, 200” fits for the 2D and 3D models are 0.76, 0.75, and 0.75 and 0.88, 0.87, and 0.85 for the 2D and 3D models, respectively.

## Discussion

In this study, we investigated the process of determining the performance plateau for an nnU-Net framework (defined as within 0.01 Dice of the maximum predicted Dice), in respect to dataset size when segmenting total kidneys and renal tumors on internal and open-sourced CT and internal T2-weighted MR images. We found the relationship of average test Dice score and data size can be approximated with an exponential-plateau model. Using this model, we found our nnU-Net model reached a test Dice plateau of 0.93 and 0.84 for our internal multiphase CT images for kidney and tumor segmentations at 54 and 125 in the training and validation set trained with fivefold cross-validation. In contrast, MR images required more images with plateaus of 0.91 and 0.74 with 122 and 389 images required in the training and validation set for kidney and tumor, respectively. The estimated plateaus for the CT and MR tumor were stable when comparing the fits on all data and on removing the 300-size dataset. Because of how the MR tumor model predicts a plateau beyond the number of observed images, as well as the demonstrated instability when removing the 300 and 250-size dataset, we are less confident in the MR tumor estimated plateau and have identified areas in which more data would be especially valuable. Regarding the KiTS21 dataset predicting tumor segmentations, the 3D model on the 240 training-validation dataset reached a maximum observed Dice performance of $0.84 \pm 0.18$ with estimated maximum achievable Dice at 0.88 requiring 440 images, 200 more than currently available in the training-validation set.

Both CT and MR datasets yielded higher than 0.88 test Dice kidney predictions with datasets as low as 50 training-validation images. We understand that kidney tissue is easier to segment compared to tumor tissue due to it often being larger and more homogenous. MR kidney tissue tended to have more variable voxel intensities (standard deviation of 37 compared to 9.64 for CT kidney). This may explain the relatively lower performance in segmentation when comparted to CT. The standard deviation of voxel intensities for MR tumor is approximately seven times greater than CT tumor voxels, possibly explaining the need for more MR images to reach comparable segmentation performance. Another reason MR tumors might require more images is that the MR dataset includes tumors from pre-operative partial nephrectomies, which are on average smaller and therefore generally more difficult to segment.

The target performance for a specific organ/tissue deep learning segmentation model has been previously established to be within inter-observer variability. This may be explained by human annotations used as the reference standard. Studies focused on CT kidney parenchyma segmentation reported inter-observer Dice scores ranging from 0.96 to 0.99 [25, 26] and for MR between 0.93 and 0.94 [27]. In the case of renal tumors, the inter-observer Dice scores reported for CT imaging range between 0.87 and 0.93 [28, 29] and in MR imaging from 0.78 to 0.87 [12]. Based on these parameters, we found that the kidney and tumor model performance presented in this study for multiphase CT and T2-weighted MR images was comparable to previously presented measures for inter-observer variability. The inclusion of the KiTS21 sub-analysis extends our work in only using corticomedullary contrast phase images as well as examining the effect of different model architectures, namely, the 2D and 3D nn-UNet models. It is interesting to note that the number required to reach dataset saturation for the 2D model is much less than the 3D model, 177 images vs. 440 images, despite it predicting lower possible Dice performance (0.76 vs. 0.89). This finding reveals that different architectures have different data efficiency and performance limits further extending the use of our method to allow investigators a standardized method to evaluate different kinds of model in real time for a task with a specific target organ and image modality. Whether due to size, heterogeneity, being paired or non-paired target structures, or other elements, different organs and/or pathology have different requirements to segment accurately as evidenced by varying validated inter-observer values [30].

Limitations to this work include different processing for the CT and MR images, the inclusion of partial nephrectomy patients in the MR dataset, and the lack of additional MR images to further assess the relationship between the dataset size and model performance. Our CT images were clipped around the kidneys presenting an easier task for automatic segmentation than the abdominal MR images. This clipping step was similar to a coarse-to-fine segmentation strategy found in the best performing KiTS21 models, where the initial model identifies the renal region of interest in the full CT abdomen and pelvis image before segmenting the specific tissue [16]. Tumors in the MR dataset were on average smaller than those in the CT dataset since the images came from patients undergoing partial and radical nephrectomies. In addition, we did not evaluate different configurations in the nnU-Net framework nor a series of different nnU-Net models as employed in some KiTS21 submissions [16, 17]. The last major limitation of this work is that this method establishes plateau performance relative to the holdout test set, which segmentation model developers must independently ensure is representative of the real-world images for the intended task. We propose our method serving to establish a good candidate for clinical application but not replacing the value of rigorously designed prospective clinical trials to ensure robustness to real-world examples.

Future potential research spawned from our present work may include the impact of including other specific renal anatomic structure and additional organs. Presumably, more renal anatomic structures would require more examples in the training and validation set to provide robust predictions. This would be especially true for structures like renal cysts which may only be present on a subset of training examples. The KiTS21 challenge included additional labels for simple cysts, providing a training set to explore this impact further. It also would be interesting to examine whether the plateau point for dataset sizes is different for different organ systems [31]. Future work to further validate this relationship can provide guidance in the curation of custom segmentation datasets for medical image segmentation purposes.

## Conclusion

An exponential-plateau model demonstrated how researchers can determine estimate how much if any benefit will be observed in final nnU-Net model performance from increasing the amount of training set data. Such application can help in the development of nnU-Net models by isolating whether sub-optimal performance may be secondary to training dataset size.

## Data Availability

Sharing of internal datasets would be a violation of HIPAA and the policies provided by our institutional review board related to this study. The external dataset 2021 Kidney and Kidney Tumor Segmentation Challenge is available in the KiTS21 repository, [https://kits21.kits-challenge.org/].
